# Stress Related Disorders and the Risk of Kidney Disease

**DOI:** 10.1016/j.ekir.2020.12.032

**Published:** 2021-01-13

**Authors:** Guobin Su, Huan Song, Vivekananda Lanka, Xusheng Liu, Fang Fang, Unnur A. Valdimarsdóttir, Juan Jesus Carrero

**Affiliations:** 1 National Clinical Research Center for Kidney Disease, State Key Laboratory of Organ Failure Research, Department of Nephrology, Nanfang Hospital, Southern Medical University, Guangzhou City, Guangdong Province, China; 2 Department of Nephrology, Guangdong Provincial Hospital of Chinese Medicine, The Second Affiliated Hospital, Guangzhou University of Chinese Medicine, Guangzhou City, Guangdong Province, China; 3 Department of Medical Epidemiology and Biostatistics, Karolinska Institutet, Stockholm, Sweden; 4 Global Health – Health Systems and Policy, Department of Public Health Sciences, Karolinska Institutet, Stockholm, Sweden; 5 European Renal Nutrition Working Group of the European Renal Association–European Dialysis Transplant Association (ERA-EDTA); 6 West China Biomedical Big Data Center, West China Hospital, Sichuan University, China; 7 Center of Public Health Sciences, Faculty of Medicine, University of Iceland, Reykjavík, Iceland; 8 Department of Environmental medicine, Karolinska Institutet, Stockholm, Sweden; 9 Department of Epidemiology, Harvard T H Chan School of Public Health, Boston, Massachusetts, USA

**Keywords:** acute kidney injury, chronic kidney disease, cohort, posttraumatic stress disorder, reaction to severe stress, SCREAM

## Abstract

**Introduction:**

Stress related disorders (SRDs, i.e., psychiatric disorders induced by significant life stressors) increase vulnerability to health problems. Whether SRDs associate with risk of acute kidney injury (AKI) and chronic kidney disease (CKD) is unknown.

**Methods:**

A population-matched cohort study in Sweden included 30,998 patients receiving a SRDs diagnosis and 116,677 unexposed patients matched by age, sex and estimated glomerular filtration rates (eGFR). The primary outcome was CKD progression, defined as a sustained relative decline in eGFR of more than 40% or commencement of kidney replacement therapy. The secondary outcome was AKI, defined by death or hospitalization attributed to AKI or rapid creatinine changes (increase ≥ 0.3 mg/d over 48 hours or 1.5x over 7 days). Cox models were used to estimate hazard ratios (HRs) with 95% confidence intervals (CIs).

**Results:**

During a medium follow-up of 3.2 years, compared to the unexposed, patients with SRDs (median age 45 years, 71% women), were at increased risk of CKD progression (HR 1.23, 95% CI 1.10-1.37) and AKI (HR 1.22, 95% CI 1.04-1.42). While the HR of CKD progression remained similarly elevated during the entire follow-up period, the association with AKI was only observed during the first year after SRDs diagnosis. Results were consistent in stratified analyses, when only considering AKI-hospitalizations/death, and when disregarding eGFR measurements close to index date.

**Conclusions:**

A diagnosis of SRDs is associated with subsequent risk of AKI and CKD progression. While studies should confirm this observation and characterize underlying mechanisms, close monitoring of kidney function following SRDs diagnosis may be indicated.

## Graphical abstract

**Figure:**
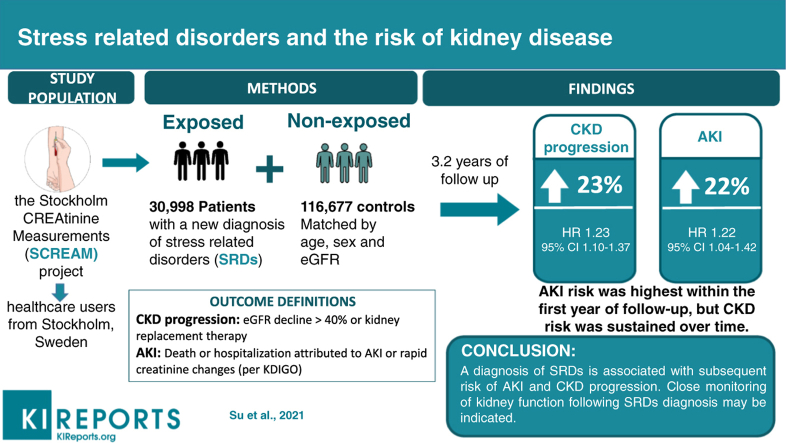

Stress related disorders (SRDs) are a group of psychiatric disorders (namely acute stress reaction, post-traumatic stress disorder (PTSD), and adjustment disorders), that appear as a consequence of excessive or prolonged psychological stress, such as the death of a loved one, a diagnosis of life-threatening illness, natural disasters, or violence. SRDs differ from other common psychiatric disorders such as depression or anxiety in its attribution to a precise psychological trauma or a stressful life event. Accumulating evidence suggests SRDs may compromise various physiological systems, increasing susceptibility to disease^1^, ^2^, ^3^, ^4^ and death,^5^ particularly among people who develop psychiatric disorders as a result of their stress.^6^^,^^7^ Animal and human studies suggest modulation of the hypothalamic-pituitary-adrenal axis^8^ and autonomic nervous system^9^^,^^10^ in response to stress, which may subsequently result in loss of humoral and cell mediated immunity,^11^ increased inflammatory reactivity,^11^ and ischemia.^3^^,^^9^

Chronic kidney disease (CKD) is a public health problem with high population prevalence,^12^ elevated healthcare costs^13^ and poor outcomes.^14^ In previous studies, exposure to SRDs has been associated to the subsequent risk of cardiovascular disease (CVD),^1^ autoimmune diseases (such as antineutrophil cytoplasmic antibodies (ANCA) vasculitis and systematic lupus erythematosus)^2^ and infections.^4^ Both CVD and autoimmune diseases are also established risk factors for kidney function decline.^15^^,^^16^^,^^17^^,^^18^ Further, psychological stressors have been associated with ischemia in the kidney,^19^^,^^20^ activated sympathetic nervous system activity,^9^^,^^10^ altered the hypothalamic-pituitary-adrenal axis,^8^ and impaired immune function.^11^ In this study, we hypothesized SRDs may primarily increase the risk of CKD progression. As a secondary analysis, we also explored the association between stress related disorders and acute kidney injury (AKI).

## Methods

### Study Design

This study was based on the Stockholm CREAtinine Measurements (SCREAM) project,^21^ a healthcare utilization cohort that contained all residents from the region of Stockholm that underwent serum creatinine testing in outpatient or inpatient care during 2006–2011. Creatinine and other laboratory data were linked with regional and national administrative databases for information on healthcare utilization (including primary healthcare), diagnoses, dispensed drugs, validated renal replacement therapy endpoints and death, with minimal loss to follow-up. The study used only de-identified data, and thus, individual informed consent was not required. The study was approved by regional institutional review boards and adhered to the Declaration of Helsinki.

### Population-Matched Cohort

#### Primary Cohort

We compiled a cohort of individuals receiving a new diagnosis of SRDs (incident diagnosis). The day of the diagnosis was considered the index date. Eligible participants for this cohort were adults (> 18 years old) receiving a new diagnosis of SRDs (10^th^ version International Classification of Diseases [ICD-10^th^] code: F43) in primary healthcare or inpatient/outpatient consultations within the laboratory data collection period of SCREAM (2007-2011) and with at least one available creatinine measurement (to estimate baseline kidney function) in an primary care or outpatient consultation within 12 months prior to the stress related disorder diagnosis. We ensured this was a new diagnosis of SRD by confirming there was no preceding SRDs diagnosis in the patient’s medical records since 1997, time in which the ICD-10^th^ version was implemented in Sweden. In case that a SRDs diagnosis was issued in both primary healthcare and outpatient-specialist, we selected the earliest diagnosis as the index date. We excluded persons with history of kidney transplantation or undergoing maintenance dialysis at time of diagnosis, as well as patients with missing information on age or sex.

To estimate baseline kidney function, we calculated estimated glomerular filtration rate (eGFR) according to CKD-EPI (CKD Epidemiology Collaboration) formula,^22^ using the mean of all available creatinine measurements undertaken in outpatient care within 12 months before the diagnosis of SRD. We did not consider inpatient creatinine measurements as they more likely reflect disease than true kidney function. We considered implausible and disregarded plasma creatinine values < 0.3 and > 17.0 mg/dl.

#### Secondary Cohort

Since the time elapsing between the occurrence of the traumatic event and the diagnosis of SRDs may vary, it is possible that the symptoms of traumatic stress may have an earlier onset than the diagnosis date of the SRDs. We here hypothesize that symptoms of traumatic stress after the traumatic event might affect kidney function. Thus, if true, eGFR values closer to the time of diagnosis might already be affected by the exposure. To account for this, we created a secondary cohort in which patient’s baseline eGFR was calculated as the average of all creatinine measurements between 6 and 12 months prior to the diagnosis date. This resulted in a lower proportion of cases, as some of them did not undergo creatinine testing during that period.

In both cohorts and for each patient with SRDs, we then randomly selected up to four control (unexposed) individuals that, on the diagnosis date of each case, had the same birth year, sex, and eGFR category (eGFR ≥ 90 ml/min/1.73m^2^; eGFR 60-89 ml/min/1.73m2, eGFR 60-30 and eGFR < 30 ml/min/1.73m^2^) within the latest 12 months (or within the 6-12 months prior to the index date of controls) and did not have a documented diagnosis of SRDs in their medical record.

### Identification of Exposure

We defined the occurrence of SRDs as the first recorded diagnosis of this disorder (F43 in ICD-10), issued in primary position during a consultation in either primary healthcare or inpatient/outpatient specialist care. SRDs were further divided into PTSD (ICD-10: F43.1), acute stress reaction (ICD-10: F43.0), adjustment disorder and other stress reactions (ICD-10: F43.2, F43.8, and F43.9). Because PTSD might initially be diagnosed as other stress related disorders (most likely as an acute stress reaction), we classified all patients receiving a diagnosis of PTSD within 1 year after their first SRDs diagnosis as patients with PTSD.

### Outcomes

The primary outcome was CKD progression, defined as the composite of a sustained relative eGFR decline of more than 40% from baseline or commencement of kidney replacement therapy (transplantation or dialysis, as ascertained by linkage with the Swedish Renal Register http://www.medscinet.net/snr/), which has complete and validated national coverage of all kidney replacement therapy cases.

The secondary outcome was the occurrence of AKI, identified by death or hospitalization attributed to ICD-10 N17 (issued in the first or second position in the hospital discharge diagnosis or death certificate), or by clinically detected transient rapid elevations of plasma creatinine according to Kidney Disease Improving Global Outcomes (KDIGO) criteria^23^ as creatinine rise ≥ 0.3 mg/d over 48 hours or more than 1.5 times over 7 days.

We followed all participants from index date until occurrence of study outcomes, emigration from the region, death, or end of follow-up (31 December 2012), whichever occurred first. There was no loss to follow-up. For unexposed individuals who got a diagnosis of SRDs during follow-up, their contribution to the unexposed group was additionally censored on the date of the SRDs diagnosis.

### Covariates

We considered as potential confounders history of severe somatic diseases, including cardiovascular diseases (myocardial infarction, congestive heart failure, peripheral vascular disease, and cerebrovascular disease), hypertension, diabetes mellitus, chronic obstructive pulmonary disease, rheumatic disease, and peptic ulcer disease, which have been associated with both CKD^16^ and SRDs.^1^^,^^2^ Presence of comorbidities were identified based on information from primary healthcare or inpatient/outpatient consultations using ICD-10 codes issued from 1997, when the 10^th^ Swedish revision of the ICD codes was implemented, until the index date (Supplementary Table).

Given that other psychiatric disorders may be diagnosed both before and after the diagnosis of SRD, we considered other psychiatric diagnoses recorded more than 3 months before the first SRDs diagnosis as “history of other psychiatric disorders”.^2^ Diagnoses of other psychiatric disorders were ICD-10: F10-F99 except for F43.

We also considered the concurrent use of certain medications including non-steroid anti-inflammatory drugs (NSAIDs)/aspirin, renin angiotensin system (RAS) inhibitors, beta-blockers, calcium channel blockers (CCB), mineralocorticoid receptor antagonists (MRA), other diuretics, selective serotonin reuptake inhibitors (SSRI) and lithium. Some of these medications were employed to further characterize the severity of comorbidities (e.g., RAS inhibitors, beta-blockers, CCB, MRA, and diuretics indicated cardiovascular disease), while others (e.g., NSAIDs, SSRI and lithium may act as confounders because they are more often prescribed to patients with SRD and carry nephrotoxic effects *per se)*. Medications were ascertained from pharmacy dispensations in any Swedish pharmacy during the six months prior to index date (see definitions in Supplementary Table S1).

### Statistical Analysis

#### Descriptive Analyses

The distribution of baseline characteristics in the exposed and unexposed groups of both primary and secondary cohorts was described using proportions (%) for categorical data, and mean and standard deviation (SD) or median and interquartile ranges (IQR) for continuous data.

#### Main Analyses

We used Cox proportional hazards models to estimate hazard ratios (HRs) with 95% confidence intervals (CIs) of outcomes associated to SRDs, using time since the index date as the underlying time scale and clustering each patient with his/her controls (stratifying by the matching identifiers of birth year, sex and eGFR strata). We then adjusted in multivariable analyses for history of other psychiatric disorders, comorbidities, and medications in a stepwise manner. As a next step, we evaluated the temporality of the association by assessing HRs in time-fixed periods (< 12 or ≥ 12 months of follow-up). We ran these analyses in both primary and secondary cohorts.

#### Subgroup Analyses (Primary Cohort Only)

We performed stratified analyses (defined *a priori)* by age (< 60, ≥ 60 years), sex (men, women), baseline eGFR category, presence/absence of diabetes, hypertension, cardiovascular disease, history of other psychiatric disorders, psychiatric comorbidities (defined as other psychiatric disorders diagnoses from 3 months before to 1 year after the first diagnosis of stress related disorders), subtype of SRDs (PTSD, acute stress reaction, adjustment disorder and other stress reactions) and source of the SRD diagnosis (primary care, outpatient and inpatient care).

#### Sensitivity Analyses (Primary Cohort Only)

First, and because more frequent healthcare utilization (hence more frequent creatinine testing) after the stress related disorder diagnosis could explain the increased rate of AKI events, we repeated our analysis redefining this outcome solely by ICD-10 diagnoses issued as primary diagnosis at a discharge of a hospitalization/death. Second, we estimated the E-value to evaluate the risk of unmeasured confounding.^24^ Conditional on the measured covariates, e-values identify the minimum strength of association that an unmeasured confounder would need to have with both the exposure and outcome, to fully explain the observed association. Third, we created 1:1 propensity score (PS) matched cohorts using all baseline characteristics in the matching, using the nearest neighbor without replacement and with a caliper width equal to 0.01 on the logit of the propensity scores.

Two-sided p-value < 0.05 was considered statistically significant, and there was no missing data. Statistical analyses were performed using STATA version 15.1.

## Results

### Baseline Characteristics

We identified 30,998 persons with an incident stress related disorder diagnosis that fulfilled our inclusion/exclusion criteria for our primary cohort. We matched them with 116,677 unexposed participants (flow chart in Supplementary Figure S1). The median age in those with stress related disorder diagnosis was 45 years. 71% of the exposed patients were women and 71.4% had normal kidney function (i.e., eGFR ≥ 90 ml/min/1.73m^2^) (Table 1). Compared to individuals unexposed to SRDs, a higher percentage of exposed patients had a history of psychiatric disorders and more frequent use of aspirin/NSAIDs, beta-blockers, selective serotonin reuptake inhibitors, and lithium use. As compared to unexposed individuals, fewer exposed patients had hypertension or diabetes.

**Table 1:** Primary cohort: Baseline characteristics in persons with incident stress related disorder diagnosis (exposed cohort) and matched controls (unexposed cohort).

Demographics	Exposed cohort (n = 30,998)	Unexposed cohort (n = 116,677)
**Age, yr (IQR)**	45 (35-56)	46 (35-57)
**Women, n (%)**	22,092 (71)	81,068 (69)
**Follow-up, yr (median, IQR)**	3.6 (2.2-4.9)	3.1 (2.0-4.5)
**eGFR, ml/min per 1.73 m**^**2**^**(IQR)**	100.5 (87.8-111.9)	100.2 (87.1-112.0)
**eGFR strata (ml/min/1.73m**^**2**^**)**		
eGFR ≥ 90, n (%)	22,132 (71.4)	82,346 (70.6）
eGFR:60-89, n (%)	7859 (25.3)	30,366 (26.0)
eGFR:30-59, n (%)	927 (3.0)	3658 (3.1)
eGFR < 30, n (%)	80 (0.3)	307 (0.3)
**Type of stress related disorders, No. (%)**		
Posttraumatic stress disorder	2033 (6.5)	
Acute stress reaction	3476 (11.2)	
Adjustment disorder	1782 (5.8)	
Other stress reaction	23,707 (76.5)	
Psychiatric comorbidity	8816 (28.4)	13,020 (11.2)
**History of other psychiatric disorders**		
History of severe somatic disease	6921 (22.3)	19,420 (16.6)
Hypertension, n (%)	7981 (25.8)	30,654 (26.3)
Diabetes Mellitus, n (%)	1814 (5.9)	8798 (7.5)
Myocardial infarction, n (%)	623 (2.0)	2791 (2.4)
Congestive heart failure, n (%)	619 (2.0)	3300 (2.8)
Peripheral vascular disease, n (%)	389 (1.3)	1821 (1.6)
Cerebrovascular disease, n (%)	1064 (3.4)	4350 (3.7)
Recent cancer (3 years), n (%)	2003 (6.5)	9696 (8.3)
Chronic obstructive pulmonary disease, n (%)	2240 (7.2)	8237 (7.1)
Rheumatic disease, n (%)	541 (1.8)	3444 (3.0)
Peptic ulcer disease, n (%)	535 (1.7)	2029 (1.7)
**Medications**		
Aspirin/NSAIDs	8498 (27.4)	30,868 (26.5)
ACEi/ARBs	3932 (12.7)	16,239 (13.9)
Mineralocorticoid receptor antagonists	320 (1.0)	1667 (1.4)
Beta blockers	4363 (14.1)	15,399 (13.2)
Other Diuretics	2360 (7.6)	9742 (8.4)
Calcium channel blockers	1838 (5.9)	7549 (6.5)
Selective serotonin reuptake inhibitors	6667 (21.5)	10,879 (9.3)
Lithium	1387 (4.5)	3847 (3.3)

ABRs, Angiotensin II receptor blockers; ACEi, Angiotensin-converting enzyme inhibitors; IQR, Interquartile range; NSAID, non-steroidal anti-inflammatory drugs.

Baseline eGFR is defined as the average of all measurements 1-12 months before index date. History of psychiatric disorders (other than stress related disorders) diagnosed from 3 months before to 1 year after the diagnosis of stress related disorders.

Of identified exposure patients, 12,429 fulfilled inclusion criteria for the secondary cohort (i.e., with baseline eGFR estimated from measurements 6-12 months before diagnosis) and were matched with 49,683 unexposed participants in a similar manner to the primary cohort (Flow chart in Supplementary Figure 1). Overall, there was a higher proportion of patients with comorbidities and with a more frequent use of medication compared to the primary cohort (Supplementary Table S3).

### Association of Stress Related Disorders with CKD Progression and AKI

#### Primary Cohort

During a median follow-up of 3.2 years, we identified 563 individuals in the exposed group (incidence rate [IR], 5.14 [95% CI, 4.73-5.58] per 1000 person-years) and 1,771 individuals in the unexposed group (IR 4.52 [95% CI, 4.31-4.74] per 1000 person-years) that progressed to CKD. We also identified 301 AKI events (IR 2.72 [95% CI, 2.44-3.05] per 1000 person-year) among exposed patients and 923 AKI events (IR 2.42 [95% CI, 2.27-2.58] per 1000 person-years) among unexposed participants (Table 2). In crude analyses, patients with SRDs appeared to be at increased risk of CKD progression and AKI (Supplementary Figure S2). After multivariable adjustment, patients with SRDs remained at a 23% (HR:1.23, 95%CI 1.10-1.37) higher risk of CKD progression and a 22% (HR 1.22, 95%CI 1.04-1.42) higher risk of AKI compared to unexposed participants. Results were similar in the 1:1 PS matched cohorts (Supplementary Table S4).

**Table 2:** Association between stress related disorders and the risk of kidney disease.

	Primary cohort (n = 147,675)		Secondary cohort (n = 62,122)	
	No. of events (incidence∗) in exposed/unexposed	Hazard ratio (95%CI)	No. of events (incidence∗) in exposed/unexposed	Hazard ratio (95%CI)
**CKD progression**				
Controlled for birth year, sex, eGFR	563(5.14)/1771(4.52)	1.24 (1.12-1.37)	284 (6.91)/317(2.03)	3.44 (2.90-4.08)
As above + history of severe somatic disease		1.24 (1.11-1.38)		1.76 (1.41-2.20)
As above + history of severe somatic disease + ongoing medications		1.23 (1.10-1.37)		1.63 (1.29-2.06)
**Acute kidney injury**				
Controlled for birth year, sex, eGFR	301(2.73)/923(2.42)	1.28 (1.11-1.46)	171(4.13)/180(1.15)	3.88 (3.08-4.90)
As above + history of severe somatic disease		1.25 (1.07-1.45)		2.12 (1.56-2.88)
As above + history of severe somatic disease + ongoing medications		1.22 (1.04-1.42)		2.05 (1.47-2.84)

In the primary cohort, baseline eGFR is defined as the average of all measurements 1-12 months before index date; In the secondary cohort, baseline eGFR is defined as the average of all measurements 6-12 months before index date.

Conditional Cox models were stratified by matching identifiers (birth year, sex, eGFR categories) and adjusted for eGFR at baseline, Comorbid history includes history of other psychiatric disorders, history of acute kidney injury, hypertension, diabetes mellitus, cancer, myocardial infarction, congestive heart failure, peripheral vascular disease, cerebrovascular disease, chronic obstructive pulmonary disease, rheumatic disease, peptic ulcer disease

Ongoing medications includes aspirin/NSAIDs, ACEi/ARBs, MRA, beta blockers, other diuretics, calcium channel blockers, selective serotonin reuptake inhibitors and lithium.

∗ per 1000 person-yr

We observed that the relative risk of AKI was highest during the first 12 months after the diagnosis of SRDs (HR, 1.32 [95%CI, 1.04-1.68]), with the association being abrogated after one year (Table 3). A similar short-term risk association, but stronger in magnitude, was observed when defining AKI events solely by primary diagnosis at hospitalization/death (HR: 1.32, 95% CI 1.04-1.68). On the contrary, we observed the increased risk of CKD progression remained beyond one year of follow up.

**Table 3:** Outcome analysis partitioning follow up during the first 12 months and beyond, showing adjusted hazard ratios with 95% confidence intervals.

Outcomes	Adjusted Hazard Ratio (95%CI)					
	Primary cohort (n = 147,675)			Secondary cohort (n = 62,122)		
**CKD progression**	*Full follow up*	*First 12 months*	*Beyond first 12 months*	*Full follow up*	*First 12 months*	*Beyond first 12 months*
No stress related disorders	Ref	Ref	Ref	Ref	Ref	Ref
Stress related disorders	1.23 (1.10-1.37)	1.11 (0.91-1.36)	1.29 (1.13-1.48)	1.63 (1.29-2.06)	1.16 (0.77-1.74)	1.94 (1.44-2.63)
**AKI**	*Full follow up*	*First 12 months*	*Beyond first 12 months*	*Full follow up*	*First 12 months*	*Beyond first 12 months*
No stress related disorders	Ref	Ref	Ref	Ref	Ref	Ref
Stress related disorders	1.22 (1.04-1.42)	1.32 (1.04-1.68)	1.14 (0.93-1.40)	2.05 (1.47-2.84)	3.55 (2.05-6.13)	1.32 (0.83-2.13)
**AKI defined solely by hospitalization/death diagnosis**	*Full follow up*	*First 12 months*	*Beyond first 12 months*	*Full follow up*	*First 12 months*	*Beyond first 12 months*
No stress related disorders	Ref	Ref	Ref	Ref	Ref	Ref
Stress related disorders	1.12 (0.88-1.42)	1.54 (1.03-2.30)	0.91 (0.67-1.23)	1.34 (0.77-2.31)	2.32 (0.53-10.13)	0.84 (0.41-1.71)

AKI, acute kidney injury; CI, confidence interval; HR, hazard ratio.

In the primary cohort, baseline eGFR is defined as the average of all measurements 1-12 months before index date; In the secondary cohort, baseline eGFR is defined as the average of all measurements 6-12 months before index date.

Conditional Cox models were stratified by matching identifiers (birth year, sex, eGFR categories) and adjusted for eGFR (continuous), history of severe somatic disease (history of other psychiatric disorders, history of acute kidney injury, hypertension, diabetes mellitus, cancer, myocardial infarction, congestive heart failure, peripheral vascular disease, cerebrovascular disease, chronic obstructive pulmonary disease, rheumatic disease, peptic ulcer disease) and ongoing medications (aspirin/NSAIDs, ACEi/ARBs, MRA, beta blockers, other diuretics, calcium channel blockers, other blood pressure medications, selective serotonin reuptake inhibitors and lithium).

**Figure 1:**
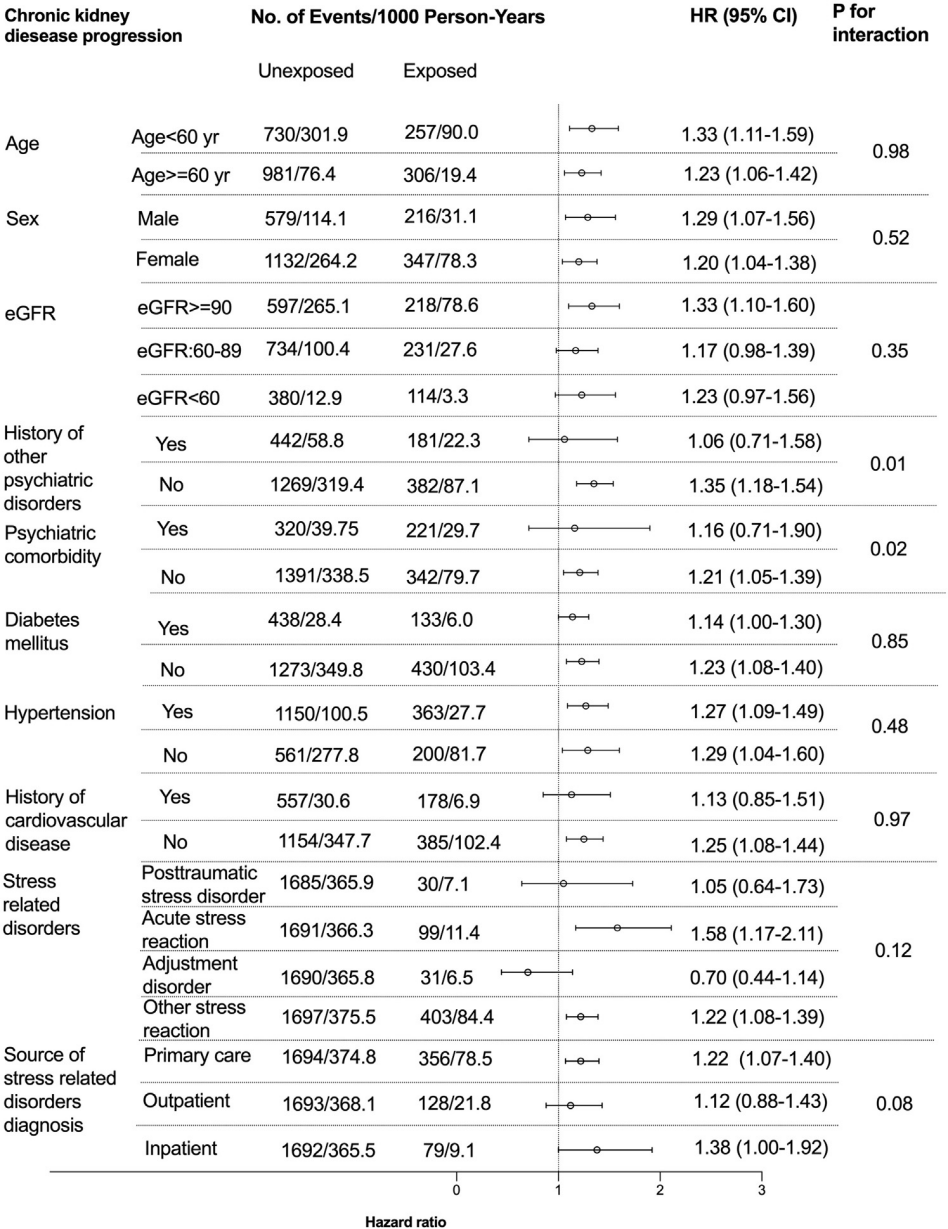
Subgroup analyses on the association between incident stress related disorders and the risk of CKD progression. History of cardiovascular disease included myocardial infarction, congestive heart failure, peripheral vascular disease, cerebrovascular disease.

**Figure 2:**
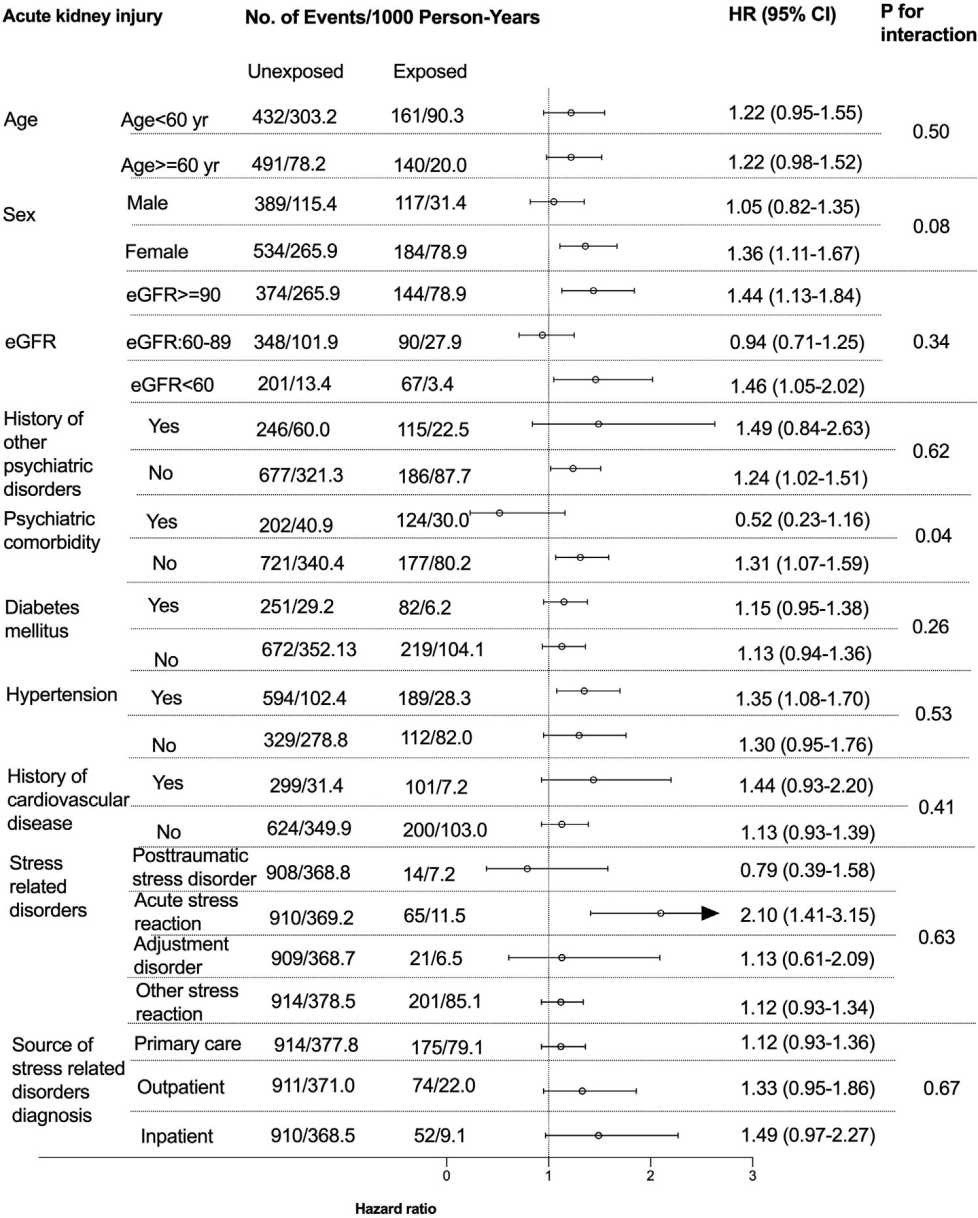
Subgroup analyses on the association between stress related disorders and AKI. History of cardiovascular disease included myocardial infarction, congestive heart failure, peripheral vascular disease, cerebrovascular disease.

Among 10 prespecified subgroups (Figure 1 and Figure 2), there was suggestion of heterogeneity with possible absence of increased CKD progression risk (HR, 1.06 [95%CI, 0.71-1.58]) in patients with history of other psychiatric disorders or AKI risk (0.52 [95%CI, 0.23-1.16]) in patients with psychiatric comorbidities. No other interactions were noted in other subgroups (age, sex, baseline eGFR categories, history of diabetes, hypertension, cardiovascular disease, types of SRD). The e-value for the outcome of CKD progression was 1.76 and 1.74 for AKI. Based on the magnitude of the HRs of other covariates with these outcomes (Supplementary Table S5 and S6), there is a moderate probability that unknown confounders may explain completely the observed associations.

#### Secondary Cohort

We identified 284 individuals in the exposed group (incidence rate [IR], 6.91 per 1000 person-years) and 317 individuals in the unexposed group (IR 2,03 per 1000 person-years) that progressed to CKD. We also identified 171 AKI events (IR 4.13 per 1000 person-year) among exposed patients and 180 AKI events (IR 1.15 per 1000 person-years) among unexposed ones (Table 2). After multivariable adjustment, patients with SRDs remained at a 63% (HR: 1.63, 95%CI 1.29-2.06) higher risk of CKD progression and a more than two folds (HR 2.05, 95%CI 1.47-2.84) higher risk of AKI compared to unexposed participants. Similarly to the primary cohort, we observed that the relative risk of AKI was highest during the first 12 months after the diagnosis of SRDs (HR, 3.55 [95%CI, 2.05-6.13]) and the increased risk of CKD progression remained beyond one year of follow up (HR, 1.94 [95%CI 1.44-2.63]) (Table 3).

## Discussion

In this study, individuals who had SRDs were at higher risk of faster CKD progression and AKI, compared to matched unexposed individuals. These associations were independent of history of other psychiatric disorders, comorbidities and medications. The risk of AKI was observed highest within the first year from the diagnosis of SRDs, but the CKD risk was sustained over time. Results were consistent across a range of sensitivity and stratified analyses, when only considering AKI-hospitalizations/death, and when disregarding eGFR measurements close to index date. This study thus identifies SRDs as a novel and plausible risk factor for kidney damage, both acute and chronic, in the community.

Our finding of an association between SRDs and the long-term risk of CKD progression adds to recent studies relating SRDs to the incidence of cardiovascular diseases, hypertension, diabetes, or obesity.^25^^,^^26^ We note that these diseases are also established risk factors for CKD. Available observational evidence pinpoints some plausible common links between SRDs and CKD. First, SRDs have been associated with the incidence of depression,^27^ and various reports consistently describe an connection between depression and the risk of developing CKD^19^^,^^20^ and end-stage kidney disease (ESKD).^28^^,^^29^ Second, case-control studies have observed that persons experiencing a stressful life event were more likely to have symptomatic kidney stones,^30^^,^^31^ which may be another mediator for subsequent CKD. Finally, stress may prompt AKI (discussed below), and this may be another link to subsequent CKD progression.^32^ The Jackson Heart Study (n = 3390) failed to observe any association between self-reported life stressors and eGFR decline or incident CKD.^33^ The discrepancy between this study and our study may be different sample sizes and studied stressors (i.e., employment, relationships, neighborhood, caring for others, legal problems, medical problems, racism and discrimination, and meeting basic needs in the Jackson Heart Study) which may differ from the clinical diagnosis of SRDs that was used in our study.

Another interesting finding of our study is the observed shorter-term association (within the first year) between SRDs and the risk of AKI. We have not been able to identify other literature on the matter. If any, a small case-control study from Brazil observed no differences in the self-report of stressful life events between patients admitted to the intensive care unit due to AKI or to other acute conditions.^34^ However, this may be a biased control population given that stressful-life events have been also linked to other acute illnesses. One possible explanation to our finding could be a higher detection of transient creatinine changes because of the more frequent healthcare use during the first months after diagnosis of SRDs. Nonetheless, it is noteworthy that the observed relative risks were still higher when we solely evaluated the more severe event of admission to hospital/death because of an AKI, and became stronger after discarding eGFR measurements performed within the 6 months prior to diagnosis. AKI may also be explained by more frequent use of lithium and SSRI among exposed patients in our study, possibly because of a higher prevalence of psychiatric comorbidity, as both these medications have been suspected to exert nephrotoxicity acutely.^35^^,^^36^ However, the association between SRDs and AKI in our study remained despite multivariable adjustment for these drugs.

The observational nature of the study precludes any inference of causality, and the novelty of our findings requires validation and expansion to other settings. However, there is some evidence supporting a biological plausibility for the associations observed, which pertain to the role of psychological stress in promoting allostatic overload, that is, the attempt of the body to find stability.^17^^,^^18^ Some of these physiological compensations relate to renal physiological effects and/or to risk factors for CKD. For instance, psychological stress induces changes in cortisol, enhances the sympathetic nervous system activity and results in sodium/water retention.^9^^,^^10^ Stress also impairs insulin sensitivity, while promotes metabolic syndrome and diabetes by altering the hypothalamic-pituitary-adrenal axis (via increased glucocorticoid and other stress hormones).^8^ Stress also increases the risk of autoimmune diseases through impairments of immune function and enhanced inflammatory reactivity.^11^ Finally, stress induces tissue ischemia and hemodynamic changes^3^^,^^9^ which may accelerate the onset of AKI. Future studies are needed to validate these findings, and well as to characterize potential underlying mechanisms.

Study strengths include the application of a population-based cohort design with complete follow-up in more than 30,000 patients diagnosed with stress related disorders. We were able to capture diagnoses from all sources of care (both primary and outpatient/inpatient specialist care). Study outcomes used both clinical diagnoses and laboratory measurements, including all creatinine testing performed in Stockholm region and thus minimizing the risk of information bias. This is important to bring upfront because many kidney events remain undiagnosed. The complete follow-up reduced selection bias due to loss of follow-up. Furthermore, the availability of rich information on comorbidities and medication use enabled considering a wide range of important confounding factors.

The main limitation of our study is that we were unable to ascertain the precise cause, intensity and timing of the life stressor that lead to the SRDs diagnosis. Some causes of SRDs (such as trauma after severe burning) may explain the subsequently observed kidney disease risks; SRDs may originate in anticipation of upcoming events (such as a risky heart operation) that may mediate the observed risks; finally, the response to the SRDs (such as prescription of SSRI, family support or unhealthy coping behaviors like heavy drinking or drugs) may also explain the associations observed. We note that controls may also be suffering from stress, but not communicate about it such as to result in a formal SRDs diagnosis; this bias, however, could be toward the null hypothesis of no difference. Despite our efforts to reduce surveillance bias (including the stricter definition of baseline eGFR in our secondary cohort, the introduction of a 1-year lag-time, the use of more stringent criteria for AKI, and estimation of e-values), we cannot rule out the possibility that distinct monitoring level between exposed and unexposed individuals has biased the observed associations, especially the short-term ones. Our median follow-up time was 3 years, which is not long enough to detect outcomes such as ESKD. Observing hazards of stronger magnitude in this secondary cohort that excludes creatinine measures prior to the diagnosis date should be considered a strength in support of our hypothesis. However, it limits generalizability; by imposing participants to have eGFR testing in the 6-12 months prior to stress related disorder diagnoses, we inevitably select sicker individuals with more frequent need of healthcare. We acknowledge the lack of information on albuminuria (which is much less often measured in routine care), on socioeconomic status (which may influence access to healthcare, family support and attitudes towards stress)^37^^,^^38^ and on air pollution (which may confound the association between stress^39^^,^^40^ and CKD^41^^,^^42^). Further studies might examine whether these factors explain or modify the association between CKD and stress. Finally, results represent Stockholm care during 2007-2011, and extrapolation to other regions, countries, ethnicities other than Caucasian and periods should be done with caution.

To conclude, this study finds that exposure to a diagnosis of SRDs was associated with the risk of AKI and CKD progression. While there is some evidence to support the plausibility behind these associations, we regard our study as hypothesis generating to foster future studies that expand these observations and characterize the plausible interplay between stress and kidney diseases. In the meantime, our results may have implications in informing clinical decisions concerning surveillance of kidney function measures during the follow up of patients with SRDs.

## Disclosures

All the authors declared no competing interests.

## Supplementary Material

Supplementary File (PDF)

**Figure S1.** Flowchart of patients eligible for the primary and secondary cohorts.

**Figure S2.** Primary cohort: Kaplan-Meier Survival Plot for the occurrence of Chronic kidney disease progression (Panel A), or acute kidney injury (Panel B) in participants with incident stress related disorders (dashed lines) or controls (straight lines).

**Table S1.**Definition of stress related disorders criteria and history of severe somatic disease.

**Table S2.** Definition of medications.

**Table S3.** Secondary cohort: Baseline characteristics in persons with incident stress related disorder diagnosis (exposed cohort) and matched controls (unexposed cohort).

**Table S4.** Sensitivity analysis: Association between SRDs and the risk of kidney disease in a 1:1 propensity score (PS) matched cohort.

**Table S5.** Primary cohort: E value and fully adjusted model in the association between stress related disorders and the risk of chronic kidney disease.

**Table S6.** Primary cohort: E value and fully adjusted model in the association between stress related disorders and the risk of acute kidney injury.
