# Supplementary material for: Stress Related Disorders and the Risk of Kidney Disease
Source: Kidney Int Rep. 2021 Jan 13;6(3):706–15. doi: 10.1016/j.ekir.2020.12.032 (PMC7938078; doi:10.1016/j.ekir.2020.12.032)
Supplement: Supplementary File (PDF) [file mmc1.pdf]

Supplementary material

Stress related disorders and the risk of kidney disease

Guobin Su, MD,PhD[1,2,3,4,5], Huan Song, MD, PhD[6,7], Vivekananda Lanka, MSc[3], Xusheng Liu, MD[2], Fang Fang, MD, PhD[8], Unnur A Valdimarsdóttir, MD, PhD[3,7,9], Juan Jesus Carrero, Pharm, PhD[3,5].

[1] National Clinical Research Center for Kidney Disease, State Key Laboratory of Organ Failure Research, Department of Nephrology, Nanfang Hospital, Southern Medical University, Guangzhou city, Guangdong Province, China

[2] Department of Nephrology, Guangdong Provincial Hospital of Chinese Medicine, The Second Affiliated Hospital, Guangzhou University of Chinese Medicine, Guangzhou city, Guangdong Province, China

[3] Department of Medical Epidemiology and Biostatistics, Karolinska Institutet, Stockholm, Sweden;

[4] Global Health – Health Systems and Policy, Department of Public Health Sciences, Karolinska Institutet, Stockholm, Sweden;

[5] European Renal Nutrition Working Group of the European Renal Association–European Dialysis Transplant Association (ERA-EDTA).

[6] West China Biomedical Big Data Center, West China Hospital, Sichuan University, China
[7] Center of Public Health Sciences, Faculty of Medicine, University of Iceland, Reykjavík, Iceland
[8] Department of Environmental medicine, Karolinska Institutet, Stockholm, Sweden
[9] Department of Epidemiology, Harvard T H Chan School of Public Health, Boston, MA, USA

Supplementary Figure S1. Flowchart of patients eligible for the primary and secondary cohorts

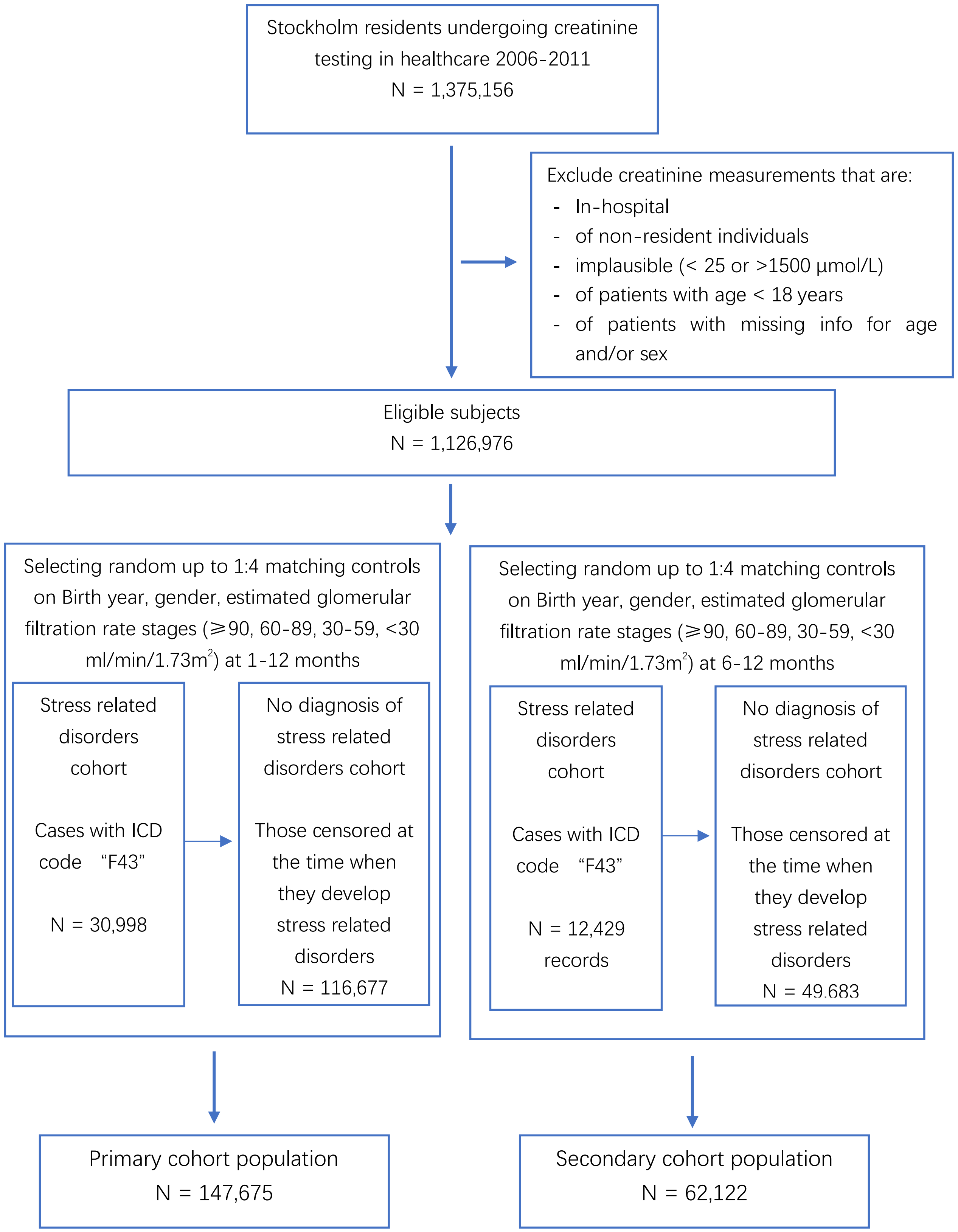

Supplementary Figure S2. Primary cohort: Kaplan-Meier Survival Plot for the occurrence of Chronic kidney disease progression (Panel A), or acute kidney injury (Panel B) in participants with incident stress related disorders (dashed lines) or controls (straight lines)

(A)

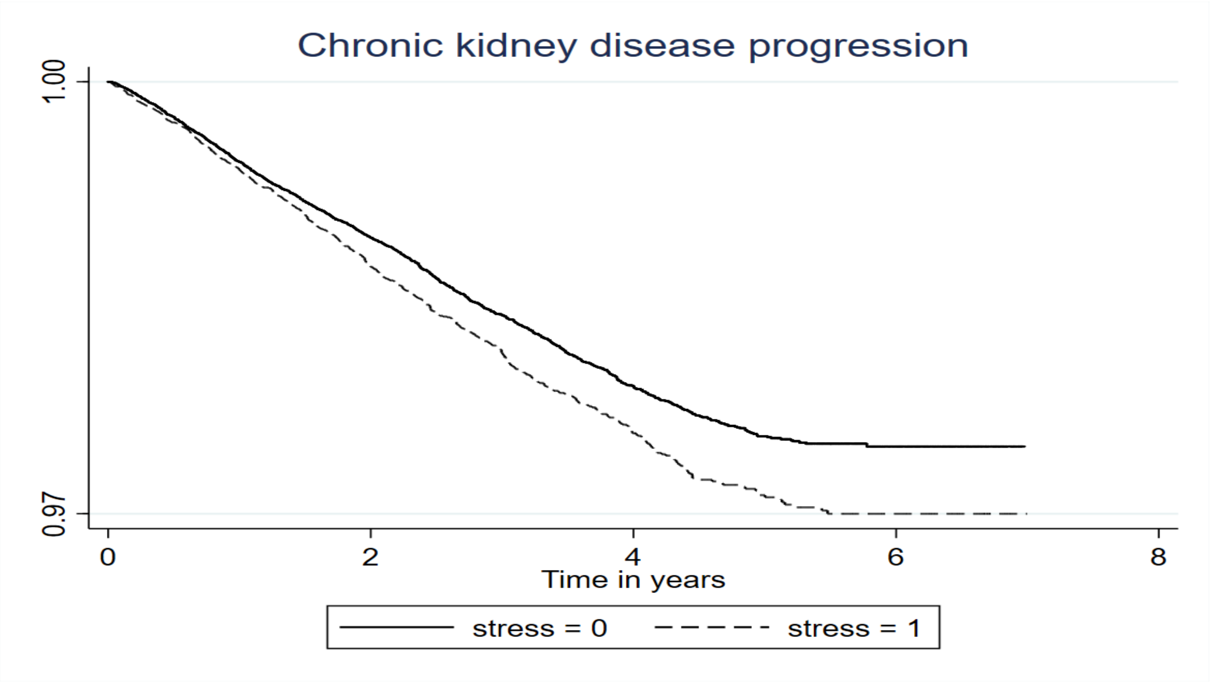

(B)

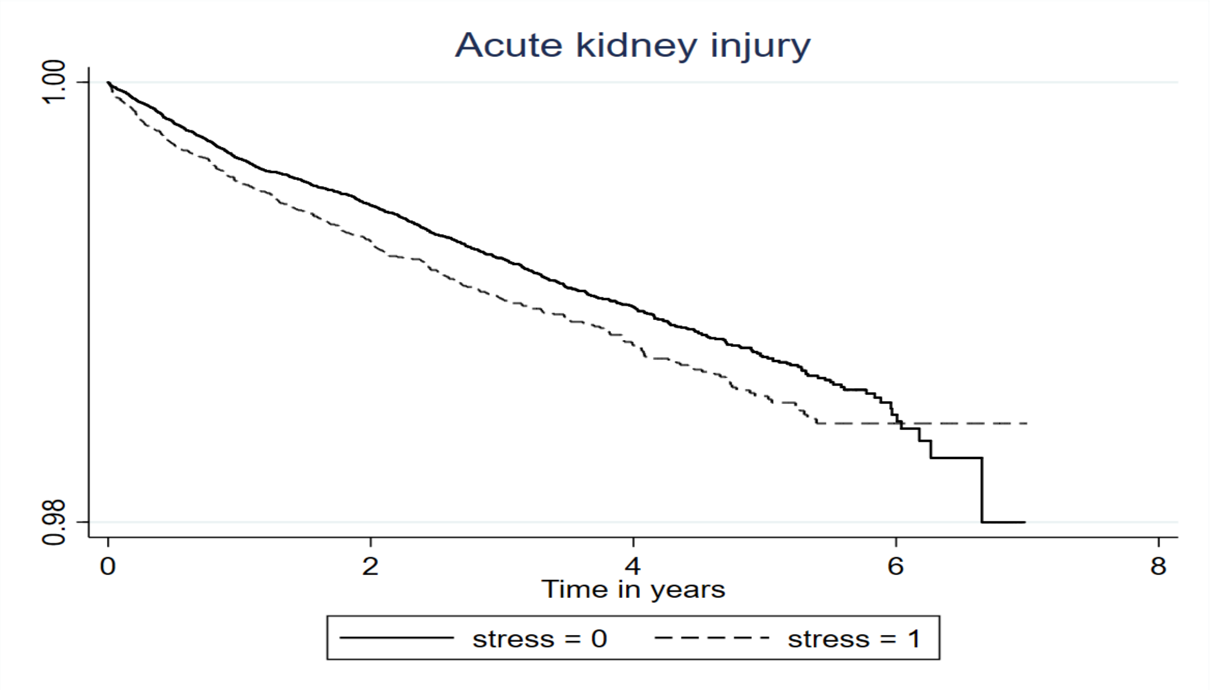

Supplementary Table S1. Definition of stress related disorders criteria and history of severe somatic disease

Conditions	ICD-10 codes	ATC codes
Disease		
All stress related disorders	F43	
PTSD	F43.1	
Acute stress reaction	F43.0	
Adjustment disorder	F43.2	
Other stress reaction	F43.8, F43.9	
history of severe somatic disease		**ATC codes**
Hypertension	I10-15	C03, C07, C08, C09
Cardiovascular disease		
Myocardial infarction	I21, I22, I23, I252	
Congestive heart failure	I099, I110, I130, I132, I255, I420, I425-429, I43, I50, P290	
Peripheral vascular disease	I70, I71, I731, I738, I739, I771, I790, I792, K551, K558, K559, Z958, Z959	
Cerebrovascular disease	G45-46, H341, I60-69	
Diabetes mellitus	E10-14	A10A, A10B
Cancer	Any malignancy, including lymphoma and leukemia (C00-C26, C30-C34, C37-C41, C43, C45-C58, C60-C76, C81-C85, C88, C90-C97) OR Metastatic solid tumor (C77-C80)	
Chronic obstructive pulmonary disease	I278, I279, J40-47, J60-67, J684, J701, J703	
Rheumatic disease	M05, M06, M315, M32-M34, M351, M353, M360	
Peptic ulcer disease	K25-K28	

ATC: Anatomical Therapeutic Classification

Supplementary Table S2. Definition of medications

Recent or current use of medications	ATC code
Aspirin/NSAIDs	B01AC06, N02BA01, M01A
ACEi/ARBs	C09A – C09D
MRA	C03DA01
Beta blockers	C07
Other Diuretics	C03
Calcium channel blockers	C08C – C08D
Lithium	N05A
Selective serotonin reuptake inhibitors	N06AB

ATC: Anatomical Therapeutic Classification; NSAID, non-steroidal anti-inflammatory drugs; ACEi: Angiotensin-converting enzyme inhibitors; ABRs: Angiotensin II receptor blockers; MRA: Mineralocorticoid receptor antagonist

Supplementary Table S3. Secondary cohort: Baseline characteristics in persons with incident stress related disorder diagnosis (exposed cohort) and matched controls (unexposed cohort)

Demographics	Exposed cohort	Unexposed cohort
Number of participants	12,429	49,683
Age, yr (IQR)	47 (36-58)	47 (37-58)
Women, n (%)	8,935 (72)	35,708 (72)
eGFR, ml/min per 1.73 m^2 (IQR)	99.4 (85.5-111.2)	99.5 (85.7-111.4)
eGFR strata (ml/min/1.73m^2)		
eGFR≥90, n (%)	8,524 (68.6)	34,096 (69.6)
eGFR:60-89, n (%)	3,337 (26.9)	13,348 (26.9)
eGFR:30-59, n (%)	520 (4.2)	2,057 (4.1)
eGFR<30, n (%)	48 (0.4)	182 (0.4)
Type of stress related disorders, No. (%)		
Posttraumatic stress disorder	951 (7.7)	
Acute stress reaction	1,403 (11.3)	
Adjustment disorder	856 (6.9)	
Other stress reaction	9,219 (74.2)	
Psychiatric comorbidity	3,929 (31.6)	2,994 (6.3)
**History of severe somatic disease**		
History of other psychiatric disorders	3,599 (29.0)	4,505 (9.1)
Hypertension, n (%)	3,808 (30.6)	5,934 (11.9)
Diabetes Mellitus, n (%)	990 (8.0)	1,882 (3.8)
Myocardial infarction, n (%)	362 (2.9)	525 (1.1)
Congestive heart failure, n (%)	380 (3.1)	623 (1.3)
Peripheral vascular disease, n (%)	239 (1.9)	347 (0.7)
Cerebrovascular disease, n (%)	578 (4.7)	853 (1.7)
Recent cancer (3 years), n (%)	1,053 (8.5)	2,194 (4.4)
Chronic obstructive pulmonary disease, n (%)	1,189 (9.6)	1,824 (3.7)
Rheumatic disease, n (%)	339 (2.7)	1,016 (2.0)
Peptic ulcer disease, n (%)	299 (2.4)	466 (0.9)
Medications		
Aspirin/NSAIDs	3,817 (30.7)	5,783 (11.6)
ACEi/ARBs	1,975 (15.9)	3,101 (6.2)
Mineralocorticoid receptor antagonists	180 (1.5)	364 (0.7)
Beta blockers	2,054 (16.5)	2,969 (6.0)
Other Diuretics	1,197 (9.6)	1,932 (3.9)
Calcium channel blockers	914 (7.4)	1,414 (2.9)
Selective serotonin reuptake inhibitors	53 (0.4)	82 (0.2)
Lithium	704 (5.7)	902 (1.8)

In the secondary cohort, baseline eGFR is defined as the average of all measurements 6-12 months before index date. IQR: Interquartile range; NSAID, non-steroidal anti-inflammatory drugs; ACEi: Angiotensin-converting enzyme inhibitors; ABRs: Angiotensin II receptor blockers; History of other psychiatric disorders (other than stress related disorders) diagnosed from 3 months before to 1 year after the diagnosis of stress related disorders

Supplementary Table S4. Sensitivity analysis: Association between SRDs and the risk of kidney disease in a 1:1 propensity score (PS) matched cohort

	Baseline eGFR defined as the average of all measurements 1-12 months before index date	Baseline eGFR defined as the average of all measurements 6-12 months before index date
	Hazard ratio (95%CI)	Hazard ratio (95%CI)
**CKD progression**		
1:1 PS matched cohort (N=53,974)	1.20 (1.04-1.39)	1.52 (1.19-1.93)
**Acute kidney injury**		
1:1 PS matched cohort (N=53,839)	1.18 (1.01-1.48)	1.54 (1.13-2.08)

We used 1:1 nearest neighbor matching between patients with and without stress related disorders. Variables included in the matching were age, sex, eGFR (continuous), history of severe somatic disease (history of other psychiatric disorders, history of acute kidney injury, hypertension, diabetes mellitus, cancer, myocardial infarction, congestive heart failure, peripheral vascular disease, cerebrovascular disease, chronic obstructive pulmonary disease, rheumatic disease, peptic ulcer disease) and ongoing medications (aspirin/NSAIDs, ACEi/ARBs, MRA, beta blockers, other diuretics, calcium channel blockers, other blood pressure medications, selective serotonin reuptake inhibitors and lithium). Matches were created without replacement using a caliper width equal to 0.01 on the logit of the propensity scores. The analysis was carried out using STATA 15.1 using module “PSMATCH2”.

Supplementary Table S5. Primary cohort: E value and fully adjusted model in the association between stress related disorders and the risk of chronic kidney disease

CKD progression	Adjusted Hazard Ratio (95%CI)	E-value (point estimate)	E-value (CI)
Stress related disorders	1.23 (1.10-1.37)	1.76	1.43
eGFR at baseline as a continuous variable in the each strata	0.99 (0.98-1.00)		
History of other psychiatric comorbidity	2.02 (1.76-2.33)		
Hypertension	1.12 (0.94-1.35)		
Diabetes Mellitus	1.70 (1.48-1.95)		
Myocardial infarction	0.99 (0.81-1.22)		
Congestive heart failure	1.26 (1.04-1.51)		
Peripheral vascular disease	1.14 (0.91-1.44)		
Cerebrovascular disease	1.08 (0.91-1.29)		
Cancer	2.14 (1.87-2.44)		
Chronic obstructive pulmonary disease	1.48 (1.26-1.73)		
Rheumatic disease	2.04 (1.63-2.55)		
Peptic ulcer disease	2.14 (1.67-2.73)		
Aspirin/NSAIDs	0.99 (0.88-1.11)		
ACEi/ARBs	1.44 (1.25-1.66)		
MRA	1.83 (1.46-2.29)		
Beta blockers	1.21 (1.05-1.39)		
Other Diuretics	1.33 (1.15-1.54)		
Calcium channel blockers	1.15 (0.99-1.34)		
Lithium	1.21 (0.94-1.55)		
Selective serotonin reuptake inhibitors	1.22 (1.05-1.42)		

Conditional Cox model stratified by matching identifiers (birth year, sex, eGFR categories) and adjusted for confounders.

Supplementary Table S6. Primary cohort: E value and fully adjusted model in the association between stress related disorders and the risk of acute kidney injury

	Adjusted Hazard Ratio (95%CI)	E-value (point estimate)	E-value (CI)
Stress-related disorder	1.22 (1.04-1.42)	1.74	1.24
History of acute kidney injury	1.10 (0.67-1.80)		
eGFR at baseline as a continuous variable in the each strata	1.00 (0.99-1.00)		
History of other psychiatric comorbidity	1.78 (1.48-2.14)		
Hpertension	1.08 (0.84-1.39)		
Diabetes Mellitus	1.88 (1.55-2.27)		
Myocardial infarction	0.77 (0.57-1.03)		
Congestive heart failure	1.49 (1.14-1.93)		
Peripheral vascular disease	1.36 (1.00-1.85)		
Cerebrovascular disease	1.05 (0.82-1.34)		
Cancer	2.56 (2.12-3.08)		
Chronic obstructive pulmonary disease	1.57 (1.26-1.97)		
Rheumatic disease	1.68 (1.25-2.27)		
Peptic ulcer disease	2.12 (1.51.-2.99)		
Aspirin/NSAIDs	1.00 (0.85-1.17)		
ACEi/ARBs	1.43 (1.17-1.75)		
MRA	1.69 (1.24-2.31)		
Beta blockers	1.35 (1.10-1.65)		
Other Diuretics	1.47 (1.20-1.82)		
Calcium channel blockers	1.01 (0.82-1.26)		
Lithium	1.79 (1.29-2.49)		
Selective serotonin reuptake inhibitors	1.24 (1.01-1.53)		

Conditional Cox model stratified by matching identifiers (birth year, sex, eGFR categories) and adjusted for confounders.
